# A New Feature Extraction Method for Ship-Radiated Noise Based on Improved CEEMDAN, Normalized Mutual Information and Multiscale Improved Permutation Entropy

**DOI:** 10.3390/e21060624

**Published:** 2019-06-25

**Authors:** Zhe Chen, Yaan Li, Renjie Cao, Wasiq Ali, Jing Yu, Hongtao Liang

**Affiliations:** 1 School of Marine Science and technology, Northwestern Polytechnical University, Xi’an 710072, China; 2 School of Physics and Information Technology, Shaanxi Normal University, Xi’an 710072, China

**Keywords:** feature extraction, improved complete ensemble empirical mode decomposition with adaptive noise, improved permutation entropy, ship-radiated noise

## Abstract

Extracting useful features from ship-radiated noise can improve the performance of passive sonar. The entropy feature is an important supplement to existing technologies for ship classification. However, the existing entropy feature extraction methods for ship-radiated noise are less reliable under noisy conditions because they lack noise reduction procedures or are single-scale based. In order to simultaneously solve these problems, a new feature extraction method is proposed based on improved complementary ensemble empirical mode decomposition with adaptive noise (ICEEMDAN), normalized mutual information (norMI), and multiscale improved permutation entropy (MIPE). Firstly, the ICEEMDAN is utilized to obtain a group of intrinsic mode functions (IMFs) from ship-radiated noise. The noise reduction process is then conducted by identifying and eliminating the noise IMFs. Next, the norMI and MIPE of the signal-dominant IMFs are calculated, respectively; and the norMI is used to weigh the corresponding MIPE result. The multi-scale entropy feature is finally defined as the sum of the weighted MIPE results. Experimental results show that the recognition rate of the proposed method achieves 90.67% and 83%, respectively, under noise free and 5 dB conditions, which is much higher than existing entropy feature extraction algorithms. Hence, the proposed method is more reliable and suitable for feature extraction of ship-radiated noise in practice.

## 1. Introduction

Ship-radiated noise contains abundant feature information of marine vessels, which can significantly improve the detection and recognition performance of passive sonar. Hence, it is of great importance to develop feature extraction techniques for ship-radiated noise [1,2,3,4,5].

Due to the influence of strong ocean ambient noise and extremely complicated acoustic medium, the received ship sound is usually nonlinear, non-stationary and highly noisy, making it a challenge to extract effective features from such a complex signal [6,7]. Many efforts have been made to search characteristics on power spectrum [3,8,9,10]. Typically, the power spectrum of a ship-radiated noise is composed of broadband spectral envelopes and narrowband spectral lines that are produced by propeller, engines, and pumps [11,12]. In practical engineering, the line-spectrum is widely adopted as a useful feature for ship recognition. There have been various spectrum-based algorithms proposed, such as the power spectrum density (PSD), the bispectrum and the higher-order spectrum [6,8,9,10]. However, the spectrum-based approaches are incapable of capturing the nonlinear characteristics of ship-radiated noise [3]. 

There have been a lot of studies proving the existence of nonlinearity in ship-radiated noise [3,4]. A recent work based on the concept of entropy has shown the effectiveness of nonlinear features in distinguishing different types of ships [7]. There are a variety of entropic algorithms in the literature including the approximate entropy [13], the sample entropy (SE) [14], and the permutation entropy (PE) [15]. Among them, PE has attracted considerable attention in many scientific and engineering fields because of its advantages of being conceptually simple and computationally fast [16,17,18]. Nevertheless, it is well known that PE encounters the following problems: (I) amplitude information of the time series is ignored [19] and (II) the entropy estimation result is liable to be affected by the equalities (i.e., equal amplitude values) in the analyzed signal [20,21]. To this end, weighted permutation entropy [19] and modified permutation entropy [21] have been presented to address problems (I) and (II), respectively. However, neither of them solves both shortcomings. To alleviate the deficiencies of PE, we proposed the improved permutation entropy (IPE) algorithm [22], which simultaneously takes amplitude information into account and eliminates the influence of equal values. In [22], it is shown that IPE outperforms PE in classifying diverse types of marine vessels. 

Signals generated from complex systems usually exhibit structures on multiple temporal scales [23]. Since all of the above mentioned entropy methods are single-scale based, they fail to account for the interrelationship of entropy and temporal scales. To remedy this, Costa et al. proposed the multiscale entropy (MSE) algorithm, in which scales are generated by the coarse-graining process [23]. The coarse-graining procedure can be combined with arbitrary entropy estimators for multiscale analysis. In our previous study [22], we also proposed a multiscale version of the IPE algorithm, termed multiscale improved permutation entropy (MIPE). 

Although MIPE has achieved promising results in ship recognition, its performance declines under noisy condition. Hence, it is of great importance to reduce noise prior to proceeding with the steps in MIPE. One way to accomplish this task is based on mode decomposition techniques, such as the variational mode decomposition (VMD) [24] and the empirical mode decomposition (EMD) [25]. Compared with VMD, EMD is data-driven and parameter-free, so it is more suitable for nonlinear and nonstationary signal analysis in practice. EMD is able to adaptively decompose a time series into a group of intrinsic mode functions (IMFs) with different central frequencies. The IMFs can be classified into three categories, namely, noise IMFs, noise-dominant IMFs, and signal-dominant IMFs. The noise reduction process is conducted by removing the noise IMFs and noise-dominant IMFs. A serious shortcoming in the EMD is the mode mixing problem, where oscillations with very disparate scales could appear in one mode [26]. In order to overcome the mode mixing problem, the ensemble empirical mode decomposition (EEMD) [26] was proposed by Wu and Huang in 2009. The EEMD algorithm performs the EMD over an ensemble of the signal plus white Gaussian noise and obtains the final results by averaging the corresponding IMFs. Even if EEMD remarkably improve the reliability of EMD, it also brings additional problems. Firstly, the reconstructed signal (i.e., the sum of IMFs) inevitably contains residual noise. Secondly, different realizations of signal plus noise may result in different number of modes, which makes it difficult for final averaging. The complementary ensemble empirical mode decomposition (CEEMD) [27] attempted to eliminate the residual noise completely by adding pairs of positive and negative white Gaussian noise, but the final averaging problem remains unsolved. To remedy this, the complementary ensemble empirical mode decomposition with adaptive noise (CEEMDAN) was proposed [28], which has been regarded as a significant improvement on EEMD. Due to its strong ability in complex signal analysis, the method becomes popular in various areas such as fault diagnosis [29], wind speed forecasting [30], seismology [31,32,33,34] and acoustic signal processing [35]. However, CEEMDAN may produce some spurious modes in the early stages of the decomposition [36,37]. Recently, this algorithm was further improved by Colominas et al., who proposed the improved complementary ensemble empirical mode decomposition with adaptive noise (ICEEMDAN) [36].

**Figure 1:**
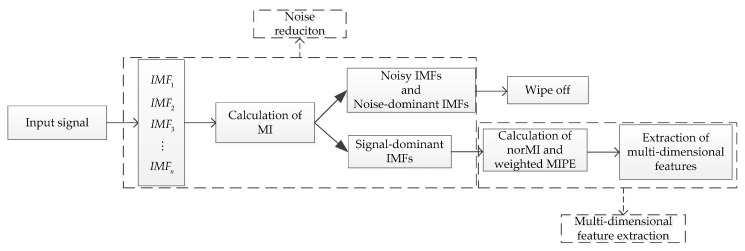
The flow diagram of the proposed method.

Since mode decomposition-based and entropy-based techniques have a lot of advantages in processing complex time series, they have attracted more and more attention in ship recognition areas. In [22], MIPE was introduced for ship classification. In [38], Li et al. proposed a feature extraction scheme based on permutation entropy of the IMF with the highest energy, where the IMFs were obtained by EMD. In [39], VMD and fluctuation based dispersion entropy (FDE) [40] were applied to analyze underwater acoustic signals, where the signal-dominant IMF was selected by calculating the FDE difference between each IMF and the original signal. However, the performance of MIPE [22] declines under noisy condition. Moreover, both EMD-PE [38] and VMD-SIMF-FDE [39] schemes are single-scale based. In the present study, a new feature extraction method for ship-radiated noise is proposed based on ICEEMDAN, norMI, and MIPE. The flow diagram of the proposed method is shown in Figure 1. In order to reduce noise and overcome the problems in EMD-based algorithms, the ICEEMDAN is firstly utilized to decompose the ship sound. Then, the noise IMFs and noise-dominant IMFs are eliminated by calculating the mutual information (MI) [41] between each IMF and the original signal. After that the norMI and the MIPE of the signal-dominant IMFs are calculated. Next, the norMI is used to weigh the corresponding MIPE result. The weighted MIPE result takes the importance of every signal-dominant IMF into account. The multiscale feature vector is finally defined as the sum of the weighted MIPE results. The proposed method inherits the advantages of both ICEEMDAN and MIPE algorithms, which significantly improves the effectiveness and reliability of existing methods for feature extraction of ship-radiated noise.

The remainder of this paper is organized as follows: the proposed feature extraction scheme is described in Section 2; simulation and experimental results are provided in Section 3 and Section 4 respectively; and the paper is concluded in Section 5.

## 2. Basic Theory

### 2.1. ICEEMDAN

**Figure 2:**
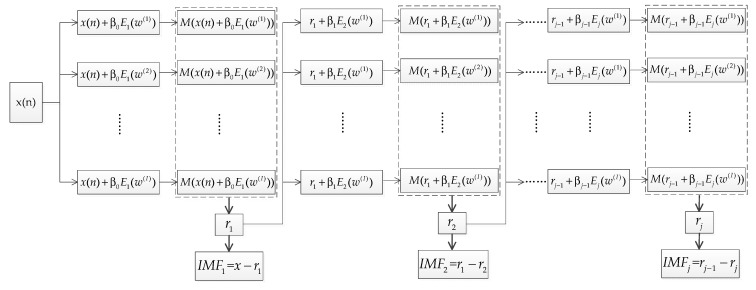
The flowchart of the improved complementary ensemble empirical mode decomposition with adaptive noise (ICEEMDAN) algorithm.

The flowchart of the ICEEMDAN algorithm is shown in Figure 2. Given a time series x(n), let $E_{k}(\cdot )$ be the *k*th IMF obtained by EMD and define M(⋅) as the operator that calculates the local mean of a signal, the ICEEMDAN algorithm is described as follows:

(1) Notice that $E_{1}(x)=x-M(x)$, for $x^{i}(n)=x(n)+\beta_{0}E_{1}(w^{\left(i\right)})$, i=1,2,⋯I, the first residue is obtained by $r_{1}=<M(x^{i}(n))>$, where <⋅> denotes the averaging operator.

(2) The first mode can be written as $IMF_{1}=x-r_{1}$.

(3) The second residue is calculated by $r_{2}=<M(r_{1}+\beta_{1}E_{2}(w^{\left(i\right)}))>$, and the second mode is expressed as $IMF_{2}{=r}_{1}-r_{2}$.

(4) For j=3,4,⋯,J, estimate the *j*th residue and the *j*th mode by $r_{j}=<M(r_{j-1}+\beta_{j-1}E_{j}(w^{\left(i\right)}))>$ and $IMF_{j}{=r}_{j-1}-r_{j}$, respectively.

(5) Step (4) is repeated until all IMFs are obtained.

Constants $\beta_{j-1}$ are selected to adjust the signal-to-noise ratio (SNR) between the residue and the added noise. For j=1, $\beta_{0}=\varepsilon_{0}std(x)/std(E_{1}(w^{\left(i\right)}))$, where std(⋅) stands for the standard deviation (SD) and $\varepsilon_{0}$ is the reciprocal of the desired SNR between the input signal x(n) and the first added noise [36]. For j≥2, $\beta_{j}=\varepsilon_{0}\mathrm{std}(r_{k})$.

### 2.2. Mutual Information and Normalized Mutual Information

The MI of two discrete random variables X and Y can be expressed as:

(1) $$MI(X;Y)=\sum_{y\in Y}\sum_{x\in X}p(x,y)\log(\frac{p(x,y)}{p(x)p(y)}),$$

where p(x) and p(y) denote the probability density function of X and Y, respectively; and p(x,y) represents their joint probability density function. Equation (1) quantifies the interdependence of X and Y [41]. If X and Y are independent, MI(X;Y)=0. Typically, the signal-dominant IMFs are correlated with the original signal, while the opposite is true for the noise IMFs and noise-dominant IMFs. Therefore, the MI can be used as an indicator to remove the noise IMFs and noise-dominant IMFs. After that, the remaining IMFs are known as signal-dominant IMFs. In order to weigh the MIPE analysis results (see Section 2.4), the norMI of each signal-dominant IMF is defined as:

(2) $$norMI_{i}=\frac{MI(IMF_{i};x)}{\sum_{l=1}^{K}MI(IMF_{l};x)},i=1,2,\cdots ,K,$$

where K denotes the number of signal-dominant IMFs.

### 2.3. MIPE

For a time series $x=\{x_{i}\}{}_{i=1}^{N}$, with a given scale factor s, the sequence is firstly processed by the coarse-graining technique, yielding a subsequence $y^{s}$, whose elements can be represented as:

(3) $$y_{i}^{s}=\frac{1}{s}\sum_{i=(j-1)s+1}^{js}x(i),\;1\leq j\leq N/s.$$

Given embedding dimension m and time delay τ, the embedding vectors of $y^{s}$ are defined as:

(4) $$Y^{s}(j)=[y_{j}^{s},y_{j+\tau }^{s},\cdots ,y_{j+(m-1)\tau }^{s}],$$

where j=1,2,⋯N/s−(m−1)τ. Next, the first column of $Y^{s}$ (i.e., $Y^{s}(:,1)$), is symbolized based on the uniform quantification (UQ) operator, which is defined as:

(5) $$UQ(u)=\{\begin{array}{cc} 0 & y_{\min}^{s}\leq u\leq \Delta \\ 1 & \Delta \leq u\leq 2\Delta \\ \vdots & \vdots \\ L-1\;(L-1) & \Delta \leq u\leq y_{\max}^{s} \end{array}.$$

In Equation (5), L is the predefined discretization level and $\Delta =(y_{\max}^{s}-y_{\min}^{s})/L$; $y_{\min}^{s}$, and $y_{\max}^{s}$ denote the minimum and maximum value of $y^{s}$, respectively. Obviously, for an input u, the UQ operator outputs an integer ranging from 0 to L−1. The symbolization result of $Y^{s}(:,1)$ can be denoted as $Sym^{s}(:,1)$. For the *k*th column of $Y^{s}$ (i.e., $Y^{s}(k,:),\;2\leq k\leq m$), $Sym^{s}(k,:)$ is obtained by:

(6) $$Sym^{s}(j,k)=Sym^{s}(j,1)+\left\lfloor (y^{s}(j,k)-y^{s}(j,1))/\Delta \right\rfloor ,1\leq j\leq N/s-(m-1)\tau .$$

Then, each row of $Sym^{s}$ is regarded as a pattern $\pi_{l}$ ($1\leq \pi_{l}\leq L^{m}$), which is similar with the PE method. Calculate the probability distribution $p_{l}$ of $\pi_{l}$, the IPE at scale s is defined as:

(7) $$IPE^{s}=-\sum_{l=1}^{h}p_{l}\ln(p_{l}),$$

where $1\leq h\leq L^{m}$. The maximum value of $IPE^{s}$ only reaches when the patterns have a uniform distribution. Therefore, $IPE^{s}$ can be normalized as:

(8) $$NIPE^{s}=IPE^{s}/\ln(L^{m}).$$

The MIPE result is finally obtained by computing the $NIPE^{s}$ with a varying scale factor.

### 2.4. The Proposed Feature Extraction Method

**Figure 3:**
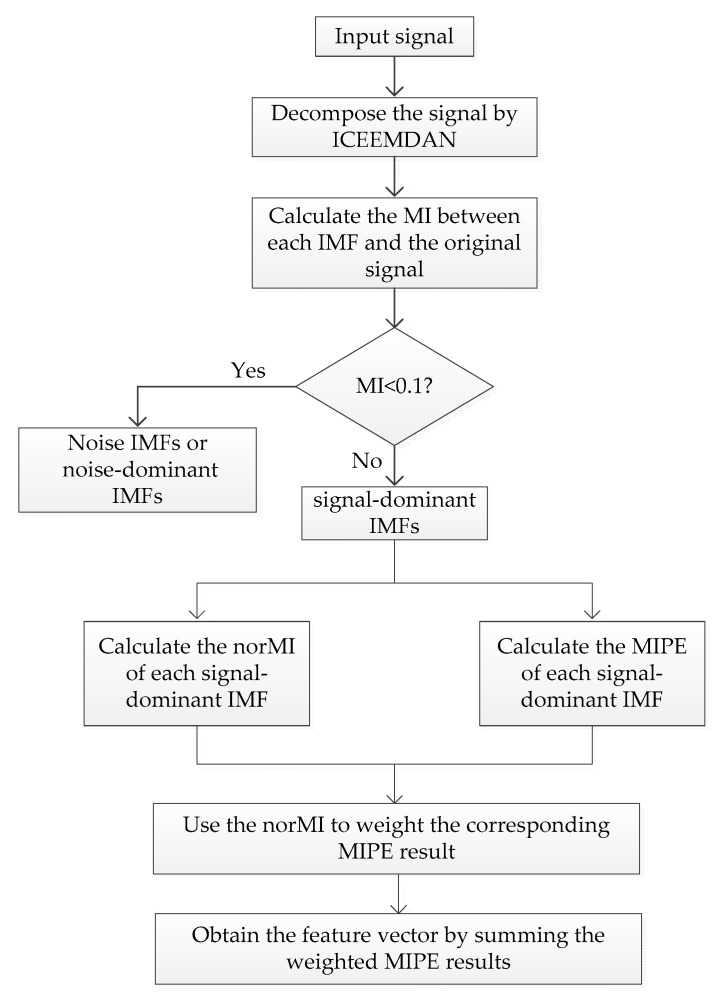
The flowchart of the proposed feature extraction method.

The detailed flowchart of the proposed feature extraction method is shown in Figure 3. The specific steps of the algorithm are as follows:

(1) The input signal is decomposed by the ICEEMDAN algorithm to obtain a group of IMFs.

(2) Calculate the MI between each IMF and the original signal, only the signal-dominant IMFs (MI≥0.1) are retained.

(3) Compute the norMI and MIPE of each signal-dominant IMF, respectively.

(4) The norMI is used as a weight coefficient to weight the corresponding MIPE result.

(5) The multi-dimensional feature vector is finally defined as the sum of the weighted MIPE results.

## 3. Simulation Results

### 3.1. Analysis of Artificial Signal Based on ICEEMDAN

In order to illustrate the advantages of the ICEEMDAN algorithm, an artificial signal was analyzed in this Section. The signal was generated by Equation (9), where w(n) denotes the white Gaussian noise with zero mean and unit variance.

(9) $$\left\{ \begin{array}{l} S(n)=s1(n)+s2(n)+\sqrt{0.001}w(n)\\ s1=\{\begin{array}{cc} 0 & 1\leq n\leq 500\\ \sin(2\pi \cdot 0.26(n-501)) & 501\leq n\leq 750\\ 0 & 751\leq n\leq 1000 \end{array}\\ s2=\sin(2\pi \cdot 0.05(n-1)) \end{array}\right.$$

**Figure 4:**
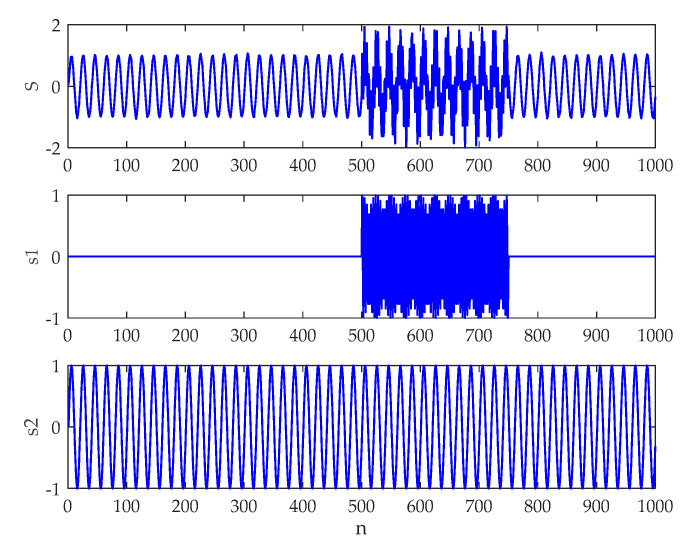
The waveform of the artificial signal.

**Figure 5:**
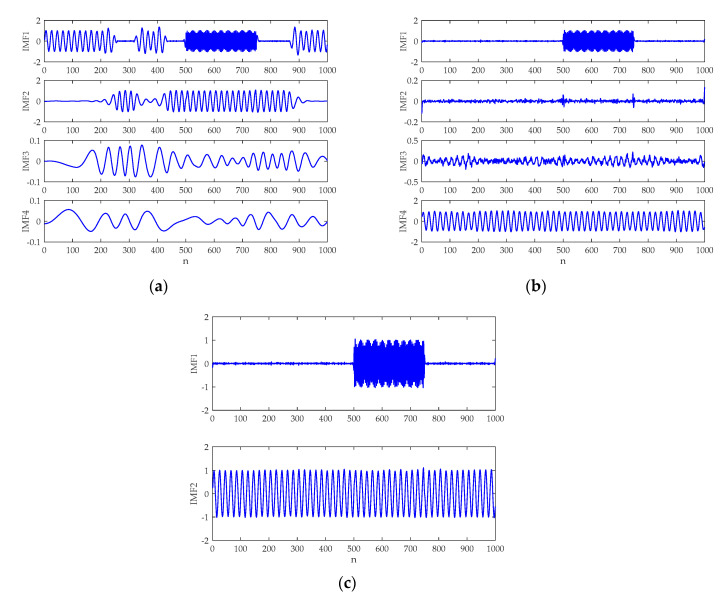
The decomposition results of the artificial signal: (**a**) empirical mode decomposition (EMD) result; (**b**) complementary ensemble empirical mode decomposition with adaptive noise (CEEMDAN) result; and (**c**) ICEEMDAN result.

The waveform of the simulation signal is demonstrated in Figure 4. The EMD, CEEMDAN, and ICEEMDAN analysis results are provided in Figure 5a–c, respectively. To perform these methods, the added noise amplitude and ensemble sizes were selected as $\varepsilon_{0}=0.2$ and I=50, respectively. Without specification, the same parameters will be used for subsequent study. Because of the local nature of the EMD method [26,27,28,36], it is evident in Figure 5a that the EMD encounters the mode mixing problem. It is also seen that the CEEMDAN produces spurious modes in IMF2 and IMF3. By comparison, the decomposition result of the ICEEMDAN algorithm corresponds well with the fact, which shows the merit of the method. Hence, ICEEMDAN would be utilized to analyze underwater acoustic signals for the first time in this paper.

### 3.2. Analysis of Artificial Signal Based on MIPE

We next applied the MIPE algorithm to analyze the autoregressive (AR) processes, which can be expressed as:

(10) $$AR_{p}(n)=\sum_{i=1}^{p}\alpha_{i}AR_{p}(n-i)+w(n).$$

**Figure 6:**
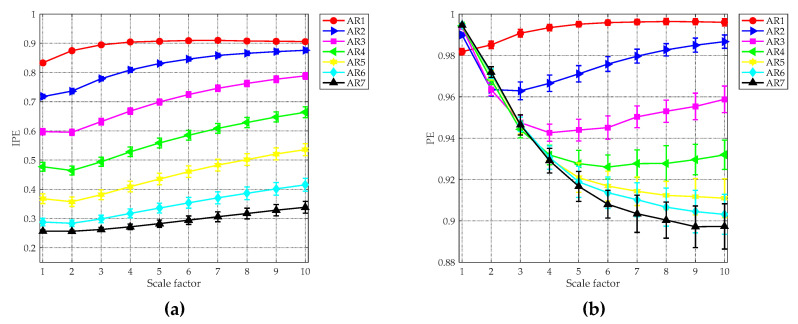
The entropy analysis results of the AR processes: (**a**) multiscale improved permutation entropy (MIPE) result; and (**b**) multiscale permutation entropy (MPE) result.

In Equation (10), p stands for the order of the AR processes, $\alpha_{i}$ represents the correlation coefficient and w(n) denotes the white Gaussian noise with zero mean and unit variance. Table 1 provides the predefined correlation coefficients for generating the AR processes with different orders. For each AR time series in Table 1, 50 independent realizations were generated. The MIPE analysis result is given in Figure 5a, where the averaged entropy values with their SD error bars are plotted. For comparison purpose, the same signals were also analyzed by the multiscale PE (MPE) [42] method, whose result is shown in Figure 6b. According to [22], the embedding dimension, time delay, data-length and discretization level were set as m=4, τ=1, N=10,000 and L=6, respectively. Without specification, the same parameters will be used for subsequent study. As can be seen, for all scale factors, the averaged IPE values decrease as the order of the AR signal increases. The result is reasonable because the AR time series with a higher order is more predictable than that with a lower order [43]. It is obvious that MIPE is able to distinguish all synthetic AR signals, whereas the PE values of $AR_{5}$, $AR_{6}$, and $AR_{7}$ are indistinguishable. The comparison result shows the strong ability of MIPE for classifying signals with different complexity. Because of this, MIPE is applied to extract entropy features of ship-radiated noise in this paper.

## 4. Experimental Results

**Figure 7:**
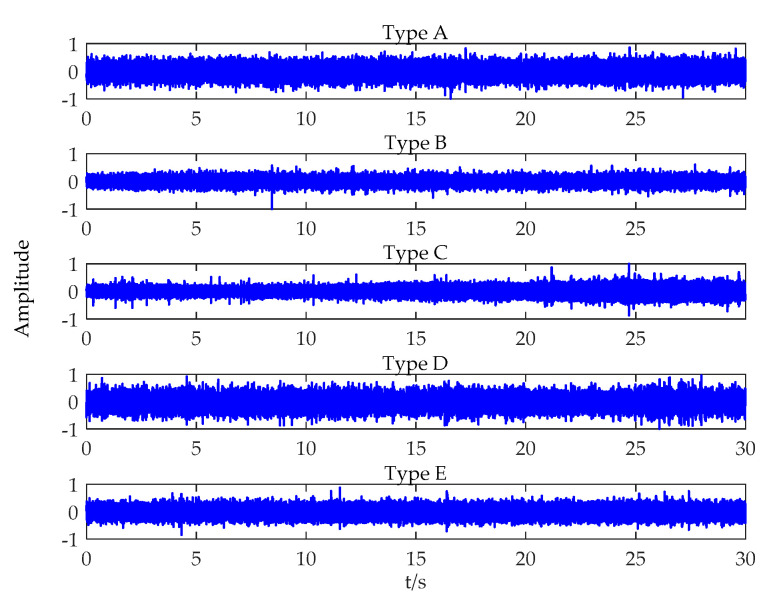
The waveforms of five types of ship-radiated noise.4.1. Feature extraction based on power spectrum density (PSD).

In this Section, the proposed feature extraction method was utilized to analyze real measured ship-radiated noise. The data sets are composed of sound samples radiated from five different ships: A, B, C, D, and E. Type A, B, and C represent a passenger ship, an ocean liner, and a motorboat, respectively. Detailed descriptions for the data can be found in [5], which is an underwater vessel noise database. Type D and E belong to a cruise ship and a ferry, respectively, and the data can be downloaded from [44]. With respect to each type of ship, we acquired 60 samples, each of which has a duration of 0.5 s. The waveforms of five types of ship-radiated noise are shown in Figure 7.

**Figure 8:**
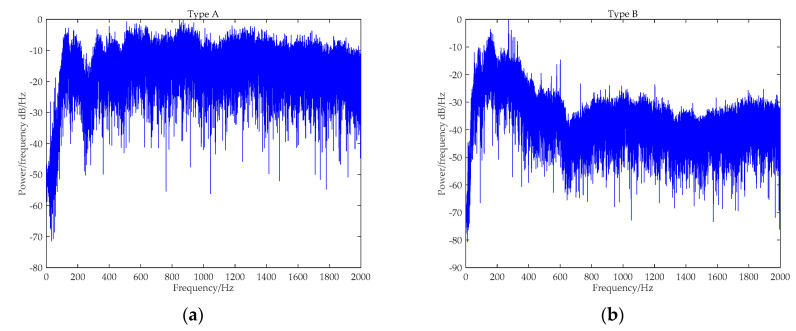

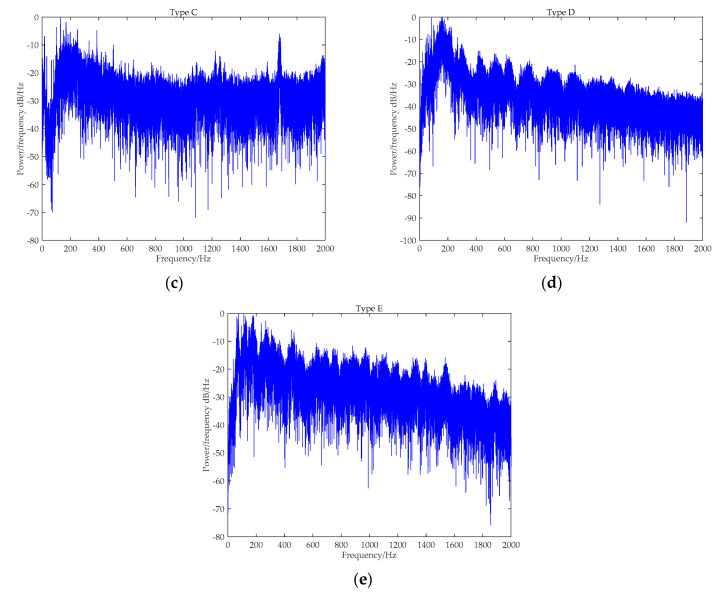
The PSD analysis results of five types of ship-radiated noise: (**a**) Type A; (**b**) Type B; (**c**) Type C; (**d**) Type D; and (**e**) Type E.

Figure 8 provides the PSD analysis results for five types of ship-radiated noise. Obviously, there are evident narrowband spectral lines existing in Figure 8b and c, where Type B and C can be easily distinguished. With regard to Type A, D, and E, no obvious spectral lines are found. Particularly, for Type D and E, there exists no evident distinction in their broadband spectral envelops. Therefore, it seems difficult to classify five kinds of ships by only using the spectrum features.

### 4.1. Feature Extraction Based on ICEEMDAN-norMI-MIPE

**Figure 9:**
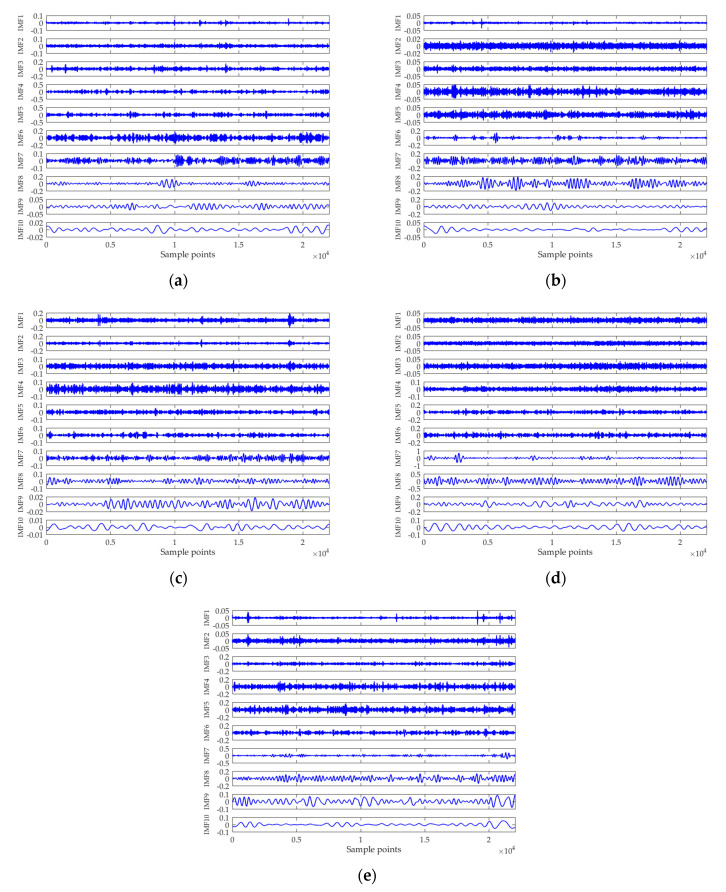
The ICEEMDAN analysis results of five types of ship-radiated noise: (**a**) Type A; (**b**) Type B; (**c**) Type C; (**d**) Type D; and (**e**) Type E.

**Figure 10:**
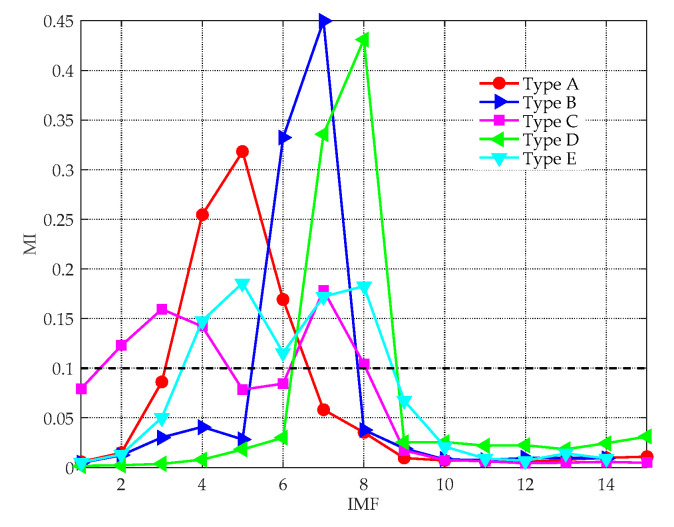
The MI between each IMF and the original signal.

**Figure 11:**
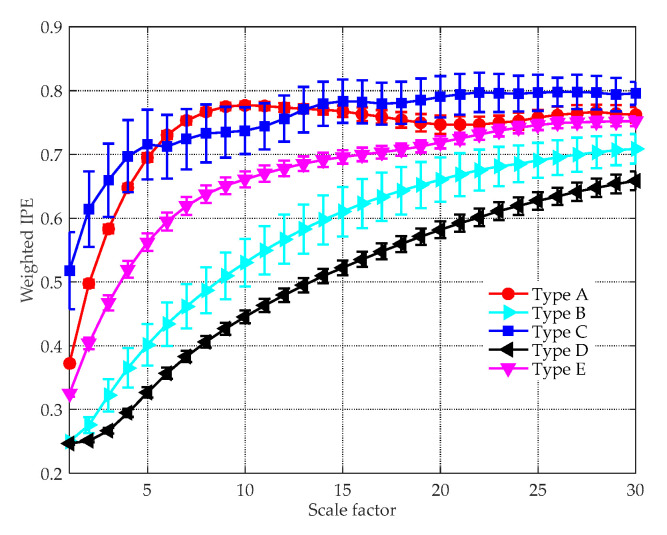
The weighted MIPE results of five types of ships.

Entropy feature can be an important supplement to existing technologies for ship classification. The effectiveness of the proposed feature extraction method was tested in this subsection. In Figure 9, a group of randomly chosen experimental data is decomposed by the ICEEMDAN algorithm, yielding a series of IMFs with different central frequencies. For clarity, only the first ten IMFs are shown in the picture. Figure 10 demonstrates the MI between each IMF and the original signal, where the black dotted line denotes the threshold to recognize the signal-dominant IMFs. After removing the noise IMFs and the noise-dominant IMFs, the norMI and the MIPE of the signal-dominant IMFs were calculated. Then the norMI was used as the weighted coefficient to weight the corresponding MIPE result. The weighted MIPE result takes the importance of every signal-dominant IMF into consideration. The multi-dimensional feature vectors of five types of ships are shown in Figure 11, where the results were obtained by averaging 60 groups of experimental data. The scale factor was selected as s=1∼30, and the error bars represent the SD of the weighted MIPE values. For different types of ship-radiated noise, there exist evident distinctions in their entropy feature vectors, suggesting that the extracted features are effective for ship classification. Moreover, the error bars remain low at all scale factors, indicating that the proposed method is reliable.

**Figure 12:**
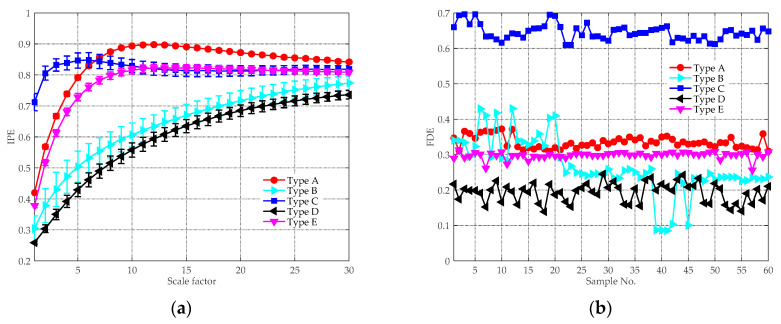
The analysis results of other feature extraction schemes: (**a**) MIPE result; and (**b**) VMD-SIMF-FDE result.

For comparison purpose, the same experimental data were analyzed by the feature extraction methods in [22] and [39]. The MIPE [22] feature extraction results are depicted in Figure 12a, where the mean IPE values with their SD error bars are plotted. Compared with Figure 11, the distinction between Type B and D are less evident as their IPE values overlap with each other, especially when s>6. This is due to that there is a lack of noise reduction procedure conducted before computing the MIPE, so the results are influenced by the ambient noise. The VMD-SIMF-FDE [39] analysis results are given in Figure 12b, where the abscissa denotes different groups of samples and the ordinate represents the FDE values. The parameters for performing the algorithm were set the same as [39]. Since the scheme is single-scale based, the interrelationship of entropy and temporal scales are discarded. As can be seen, the FDE values of Type A and E are very close. Moreover, the results of Type B overlap with other types, making them difficult to be distinguished.

**Figure 13:**
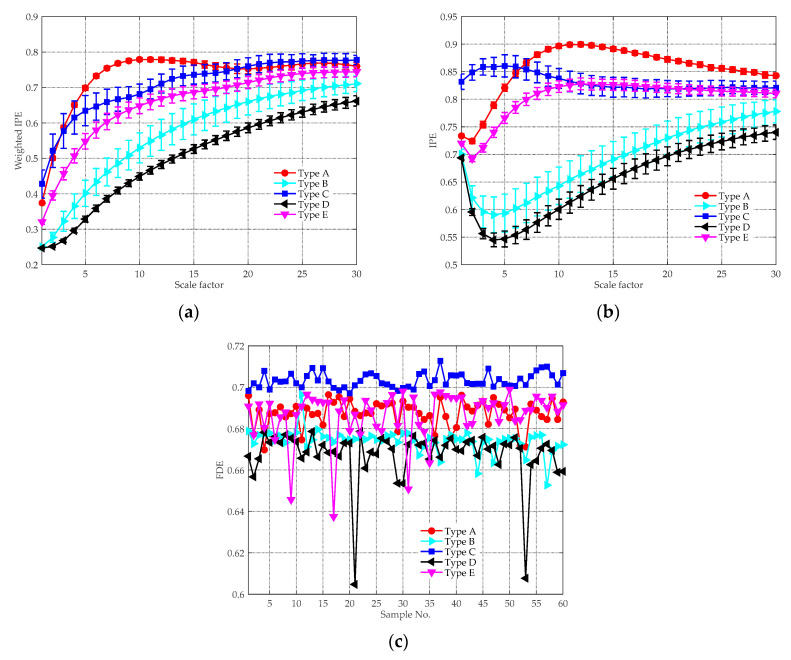
The feature extraction results under 5 dB condition: (**a**) ICEEMDAN-norMI-MIPE result; (**b**) MIPE result; and (**c**) VMD-SIMF-FDE result.

It is also interesting to compare the effectiveness of above feature extraction methods in noisy environment. To this end, white Gaussian noise was added into the ship sound to generate different SNR conditions. Figure 13 provides the feature extraction results under the 5 dB condition. Compared with Figure 11 and Figure 12, the single-scale based VMD-SIMF-FDE scheme becomes invalid due to the interference of noise. Similarly, the performance of MIPE sharply declines especially when s=1∼5. By contrast, except for Type C, the added noise has little influence to the proposed method, which further proves its reliability.

### 4.2. Ship Classification

To evaluate the performance of the above mentioned feature extraction methods quantitatively, a widely used classifier, known as the probability neural network (PNN) [45], was utilized to process the extracted features in Section 4.1. For each type of ship, 30 randomly selected noise free samples were used for training and the remaining 30 samples were for testing. Regarding the situation under noisy condition, all 60 groups of features were set as test datasets. The classification results are shown in Table 2, which corresponds well with the feature extraction results in Section 4.1. For a clean signal, the proposed method achieves a recognition rate of 90.67%, which is 8% and 13.34% higher than MIPE and VMD-SIMF-FDE, respectively. As the SNR decreases to 5 dB, the classification accuracy of the ICEEMDAN-norMI-MIPE declines to 83%, while that of the MIPE and VMD-SIMF-FDE drops to 67.33% and 20%, respectively. When the SNR further decreases to 0 dB, both MIPE and VMD-SIMF-FDE algorithms become invalid as only 20% samples are correctly classified by them. By contrast, the proposed method still has a recognition rate of 64.33%.

## 5. Conclusions

In order to extract useful features from ship-radiated noise, a new feature extraction method is proposed based on ICEEMDAN, norMI, and MIPE. The ICEEMDAN algorithm overcomes the mode mixing problem of EMD and eliminates the spurious modes in CEEMDAN. Thus, it is utilized to analyze underwater acoustic signals for the first time. Compared with PE, MIPE shows a stronger ability at classifying signals with different complexity. Therefore, it is applied to extract entropy features of ship-radiated noise in this paper. The proposed feature extraction scheme inherits the advantages of both ICEEMDAN and MIPE algorithms. In comparison of the existing feature extraction methods for ship-radiated noise, the ICEEMDAN-norMI-MIPE scheme is multi-scale based and it reduces noise prior to calculating the MIPE. Moreover, it uses norMI to weight the MIPE results, which takes the importance of every signal-dominant IMF into consideration. The validation of the proposed algorithm is proved by ship classification experiment. The proposed method obtains a recognition rate of 90.67% under noise free condition, which is 8% and 13.34% higher than the MIPE and VMD-SIMF-FDE methods, respectively. Furthermore, the classification accuracy of the proposed scheme reaches 83% under 5 dB condition, while that is only 67.33% and 20% for the MIPE and VMD-SIMF-FDE algorithms, respectively. When the SNR further decreases to 0 dB, both MIPE and VMD-SIMF-FDE algorithms become invalid. By contrast, the proposed method still has a recognition rate of 64.33%. Therefore, the proposed algorithm is more reliable and suitable for entropy feature extraction of ship-radiated noise in practice. Our method can be a supplement to existing technologies for ship classification.

## Figures and Tables

**Table 1:** The predefined correlation coefficients for generating autoregressive (AR) processes with different orders.

	$\alpha_{1}$	$\alpha_{2}$	$\alpha_{3}$	$\alpha_{4}$	$\alpha_{5}$	$\alpha_{6}$	$\alpha_{7}$
$AR_{1}$	0.5	-	-	-	-	-	-
$AR_{2}$	0.5	0.25	-	-	-	-	-
$AR_{3}$	0.5	0.25	0.125	-	-	-	-
$AR_{4}$	0.5	0.25	0.125	0.0625	-	-	-
$AR_{5}$	0.5	0.25	0.125	0.0625	0.0313	-	-
$AR_{6}$	0.5	0.25	0.125	0.0625	0.0313	0.0156	-
$AR_{7}$	0.5	0.25	0.125	0.0625	0.0313	0.0156	0.0078

**Table 2:** Recognition accuracy for four types of ships by probability neural network (PNN).

	Clean Signal	5 dB	0 dB
The proposed method	90.67%	83%	64.33%
MIPE [23]	82.67%	67.33%	20%
VMD-SIMF-FDE [39]	77.33%	20%	20%
